# Craniofacial integration and modularity in untreated cleft lip and palate

**DOI:** 10.1007/s00784-025-06296-3

**Published:** 2025-03-31

**Authors:** Sariesendy Sumardi, Anne Marie Kuijpers-Jagtman, Benny S. Latief, Hans L. L. Wellens, Piotr S. Fudalej

**Affiliations:** 1 Department of Orthodontics, Faculty of Dentistry, Universitas Indonesia, Jakarta, Indonesia; 2 Faculty of Dentistry, Universitas Indonesia, Jakarta, Indonesia; 3 Department of Orthodontics, University Medical Center Groningen, University of Groningen, Groningen, The Netherlands; 4 Department of Orthodontics and Dentofacial Orthopedics, School of Dental Medicine/Medical Faculty, University of Bern, Bern, Switzerland; 5 Department of Oral-Maxillofacial Surgery, Faculty of Dentistry, Universitas Indonesia, Jakarta, Indonesia; 6 Private Practice, Brugge, Belgium; 7 Department of Orthodontics, Jagiellonian University in Kraków, Kraków, Poland

**Keywords:** Cleft lip, Cleft palate, Geometric morphometrics, Adult, Cephalometry, Maxillofacial development

## Abstract

**Objectives:**

To quantify craniofacial variation, integration, and modularity in untreated adults with orofacial clefts who had not undergone surgery, as well as in unaffected controls.

**Materials and methods:**

Fourteen cephalometric landmarks depicting the skull base, maxilla, and mandible were identified on lateral cephalograms of 295 adult Proto-Malayid individuals. The sample included 243 individuals with unoperated clefts—179 with complete unilateral cleft lip and alveolus (UCLA, mean age 23.7 years) and 66 with complete unilateral cleft lip, alveolus, and palate (UCLAP, mean age 24.5 years)—and 50 unaffected controls (NORM, mean age 21.2 years). Geometric morphometrics were used to analyze craniofacial shape variability, integration, and modularity. Principal component analysis (PCA) was used to assess shape variability, while canonical variates analysis (CVA) was used to evaluate group differences by calculating Mahalanobis and Procrustes distances. Integration and modularity were tested for five scenarios: (1) skull base vs. maxilla vs. mandible, (2) skull base with maxilla vs. mandible, (3) skull base with mandible vs. maxilla, (4) skull base vs. maxilla with mandible, and (5) anterior vs. posterior modules. The RV coefficient and covariance ratio were used to assess covariation strength.

**Results:**

The first 6 principal components (PC1-PC6) explained 72% of the total shape variability, with vertical shape variation and sagittal relationships being the primary sources of variability. Craniofacial shape varied significantly among the groups, with the largest Mahalanobis and Procrustes distances observed between the NORM and UCLAP groups (*p* < 0.001), and the smallest between the UCLA and UCLAP groups (*p* < 0.001). Modularity and integration patterns differed between cleft-affected individuals and controls; Those with clefts had anterior and posterior modules separated by the pterygomaxillary plane, while controls showed distinct modules for the skull base, maxilla, and mandible or combined skull base-mandible and maxilla.

**Conclusions:**

Unoperated unilateral UCLA and UCLAP affect craniofacial integration and modularity.

**Clinical relevance:**

These insights highlight the importance of individualized treatment approaches that consider congenital craniofacial organization, potentially improving long-term functional and aesthetic outcomes.

**Supplementary Information:**

The online version contains supplementary material available at 10.1007/s00784-025-06296-3.

## Introduction

Craniofacial development is a highly coordinated and complex process involving interactions between various embryonic structures, tissues, and specialized cells following a cephalocaudal gradient. This process results in the formation of an integrated yet modular craniofacial complex [1]. In other words, the human craniofacial region exhibits overarching morphological integration as a coordinated structural–functional unit because its parts develop and function jointly [2–5]. However, this integration is not uniform; it is structured into semiautonomous developmental and functional modules with varying levels of integration within and between them [5–7].

Orofacial clefts (OFC) develop early in pregnancy, typically between the 5th and 10th week of gestation. These clefts result from incomplete fusion of the tissues that make up the lip and palate during embryonic development [8]. OFC are usually corrected surgically shortly after birth. Consequently, the craniofacial growth abnormalities frequently observed in patients with OFC result from a combined effect of congenital growth deficiency and surgical iatrogenesis. Determining the proportion of the effect attributable to congenital issues versus surgical intervention is challenging in humans because almost all infants undergo surgical treatment.

Recent systematic reviews, including growth studies in untreated OFC [9, 10], suggest that growth inhibition due to surgical iatrogenesis likely substantially overshadows cleft-related inborn growth deficiency. Given the limitations of investigations of untreated patients with OFC, such as relatively small sample sizes and heterogeneity in cleft type or severity, ethnicity, and age, it is impossible to establish the strength of the association, if any, between the presence of OFC and subsequent growth disturbance. Nevertheless, investigations of untreated OFC using geometric morphometric methods [11, 12] have demonstrated differences in craniofacial morphology compared to noncleft controls. These different patterns of morphological variability suggest that patients with OFC have intrinsic growth impairments that can affect future facial development. It is not clear, however, whether and to what degree OFC influence craniofacial integration and modularity. Therefore, the aim of this study was to test the hypothesis that compared with noncleft controls, untreated unilateral cleft lip and alveolus (UCLA) and untreated unilateral cleft lip, alveolus, and palate (UCLAP) affect craniofacial integration and modularity.

## Materials and methods

### Sample

This study received ethical permission from the Bioethics Committee of Universitas Indonesia, Jakarta (reference number: 1/EthEx/FKGUI/II/2015).

The facial morphology of 245 adult subjects who exhibited non-syndromic unoperated complete unilateral cleft lip and alveolus (UCLA *N* = 179, mean age 23.7 years, SD = 10.9; males *N* = 103, females *N* = 76) or non-syndromic unoperated complete unilateral cleft lip, alveolus, and palate (UCLAP *N* = 66, mean age 24.5 years, SD = 10.5; males *N* = 37, females *N* = 29) was compared with that of 50 unaffected controls (NORM mean age 21.2 years, SD = 3.2; males *N* = 25, females *N* = 25) who shared the same ethnic background. The samples were collected between 1986 and 1997 in the province of Nusa Tenggara, Indonesia, as part of a larger study [13].

The control group comprised 50 individuals without cleft conditions who volunteered from the city of Kupang, the capital of the province East Nusa Tenggara. The inclusion criteria for this group were Proto-Malayid origin, the absence of clefts or other craniofacial anomalies, no occurrence of clefts in the family history, and a normal occlusal relationship (Angle Class I). The exclusion criteria included prior orthodontic or surgical treatment in the maxillofacial region and age younger than 14 years.

### Data acquisition and analysis

Cephalometric radiographs were made with a mobile radiographic setup, incorporating a cephalostat with a focus-film distance of 1.70 m. Due to field conditions encountered during imaging of cleft and noncleft subjects, maintaining a constant radiographic magnification factor was challenging. Consequently, this study focused solely on evaluating facial shape, omitting information regarding size. Difficulties in visualizing soft tissue contours arose from the suboptimal quality of certain radiographs; therefore, the analysis of facial morphology is limited to hard tissues.

**Fig. 1:**
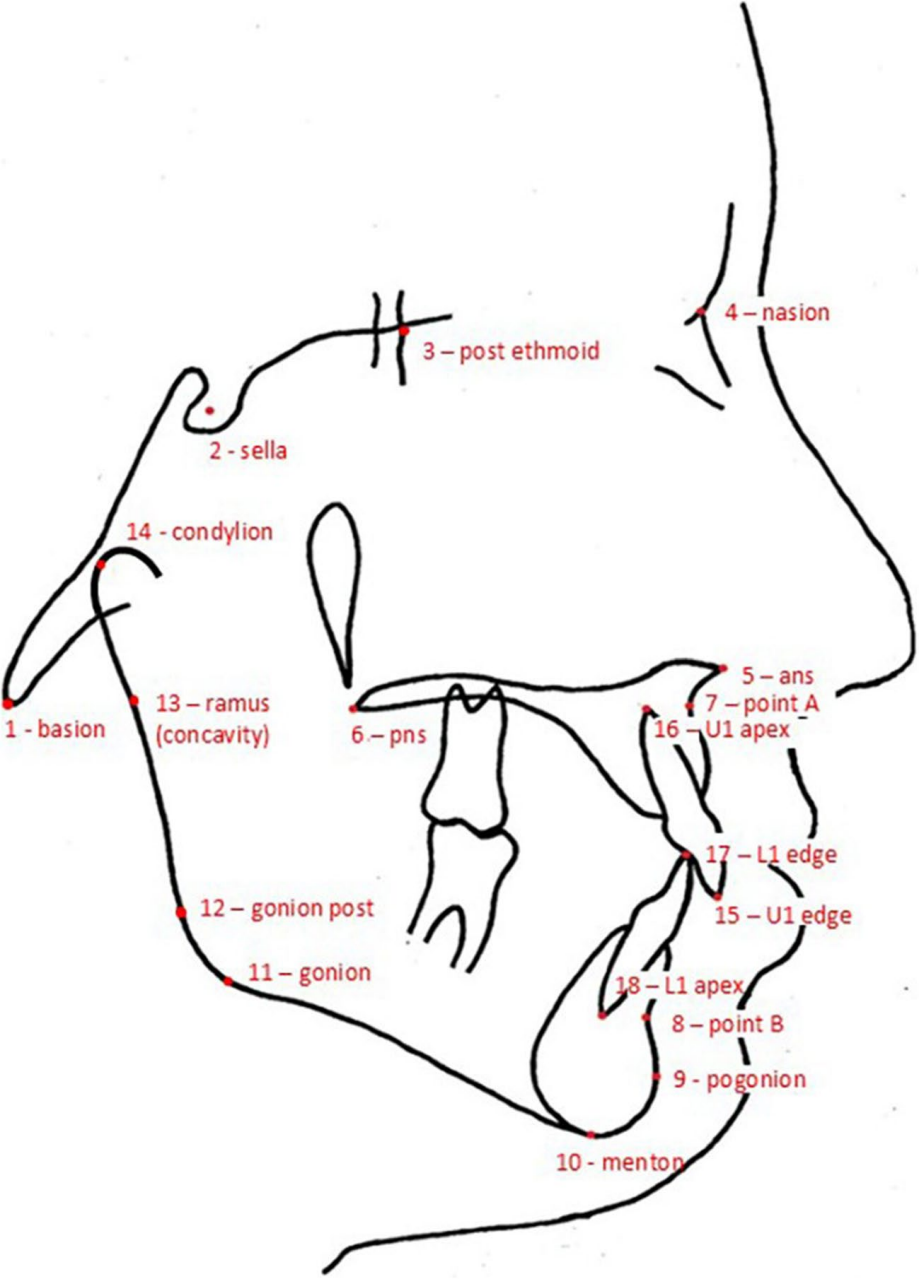
Tracing of a cephalogram indicating the anatomical landmarks that were used in the study

The cephalometric radiographs were scanned at a resolution of 300 dpi. The geometry of the cranial base, maxillary complex, mandible, and anterior dentition was captured using 14 anatomical landmarks (Fig. 1) depicting the skull base, maxilla, and mandible. These landmarks align with the studies of Latif et al. [12] and Bartzela et al. [14] on patients with bilateral clefts. A single investigator (PF) digitized the landmarks using the tpsDig2 program, version 2.18 (http://life.bio.sunysb.edu/morph/). Two-dimensional landmark coordinates were exported in TPS format for subsequent analysis using the MorphoJ program, version 1.06d [15].

### Statistical analysis

Shape variability, inter-group differences, integration and modularity were analyzed using geometric morphometrics [16]. Shape variability was scrutinized using principal component analysis (PCA). To assess intergroup differences, the Mahalanobis and Procrustes distances between group means were examined through canonical variates analysis (CVA). CVA is a method employed to identify the shape features that most effectively distinguish among multiple groups of subjects [16].

**Fig. 2:**
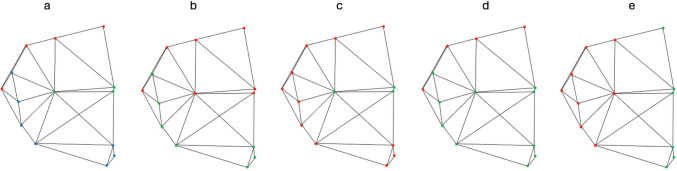
Pruned adjacency graphs, created through Delaunay triangulation (Klingenberg 2009), to illustrate all the scenarios. **a** illustrates the structural subdivision employed in scenarios with three modules; **b**-**d** illustrate the structural subdivisions employed in scenarios with two modules; and (**e**) portrays the subdivision when testing the counterpart principle

The modularity hypothesis was examined following the methodology outlined by Klingenberg [6]. The scenarios considered include one involving three modules (skull base vs. maxilla vs. mandible), and four involving two distinct modules (skull base with maxilla vs. mandible, skull base with mandible vs. maxilla, skull base vs. maxilla with mandible, as well as anterior vs. posterior craniofacial columns of the counterpart hypothesis). The five modularity scenarios were selected to investigate whether modularity is expressed rather “conventionally” along the anatomically distinct skull base, mandible and maxilla (or combinations thereof), as opposed to functionally (the counterpart hypothesis). A pruned adjacency graph, created through Delaunay triangulation [6], was utilized for all the scenarios. This graph was constructed in advance, with connections avoiding discontinuous skeletal tissue. Figure 2a illustrates the structural subdivision employed in scenarios with three modules, Figs. 2b-d illustrate the structural subdivisions employed in scenarios with two modules, and Fig. 2e portrays the subdivision used when testing the counterpart principle.

The (multi)RV coefficient [6] and covariance ratio (CR) [17] were calculated for each scenario, enabling a comparison with the corresponding values obtained from randomly generated alternative subdivisions into two to three spatially continuous modules. These alternative modules, featuring the same number of points as their counterparts in the proposed subdivision, were generated using the adjacency graph as a tool to ensure spatial continuity. Each round of Generalized Procrustes Analysis superimposition employed the simultaneous-fit approach, maintaining the relative size among the modules. The multi-RV coefficient was used for scenarios involving three modules, while the original RV coefficient was utilized for those with two modules. The CR was calculated for all scenarios. CR = 1 indicates no modularity because within- and between-module covariances are equal, while CR ≠ 1 can imply modularity because between-module covariances are smaller/larger than within-module covariances.

To assess the significance of the findings, all possible continuous alternative subdivisions were generated and compared with their respective proposed modularity scenarios. The number of alternative subdivisions exhibiting a multi-RV coefficient or CR lower than the proposed one was recorded as the P value for the statistical significance of the observed pattern. For CR, p value < 0.01 denoted statistical significance after Bonferroni correction.

To evaluate intra-observer reliability, a set of 28 images (14 with a cleft and 14 non-cleft controls) was randomly chosen and digitized again by the same investigator (PF) after a minimum interval of 1 month. The random error was quantified as the Procrustes distance between the digitizations in shape space relative to the total shape variance.

All analyses and statistical tests were performed in MorphoJ and PAST v.3 software (Øyvind Hammer, University of Oslo, Norway). Permutation tests (100,000 permutation runs) with a significance level of 0.05 were used to establish intergroup differences in facial shapes. Facial shape changes were visualized using wireframes constructed in MorphoJ.

## Results

### Method error

The observed measurement error was acceptable, accounting for 7.29% of the total variation.

### Shape variability

Principal components 1 through 6, each accounting for at least 5% of the variance, explained 72% of the variance among individuals (Appendix). The first major axis of shape variability (PC1, 26.2% of variance) demonstrated variability mainly in the vertical direction, while PC2 (15.5% of variance) described variability in the shape of the mandibular angle and horizontal position of the maxilla; PC3 (12.6% of variance) depicted variability in the size and/or position of the maxilla and mandible relative to each other and to the cranial base.

### Intergroup differences

**Fig. 3:**
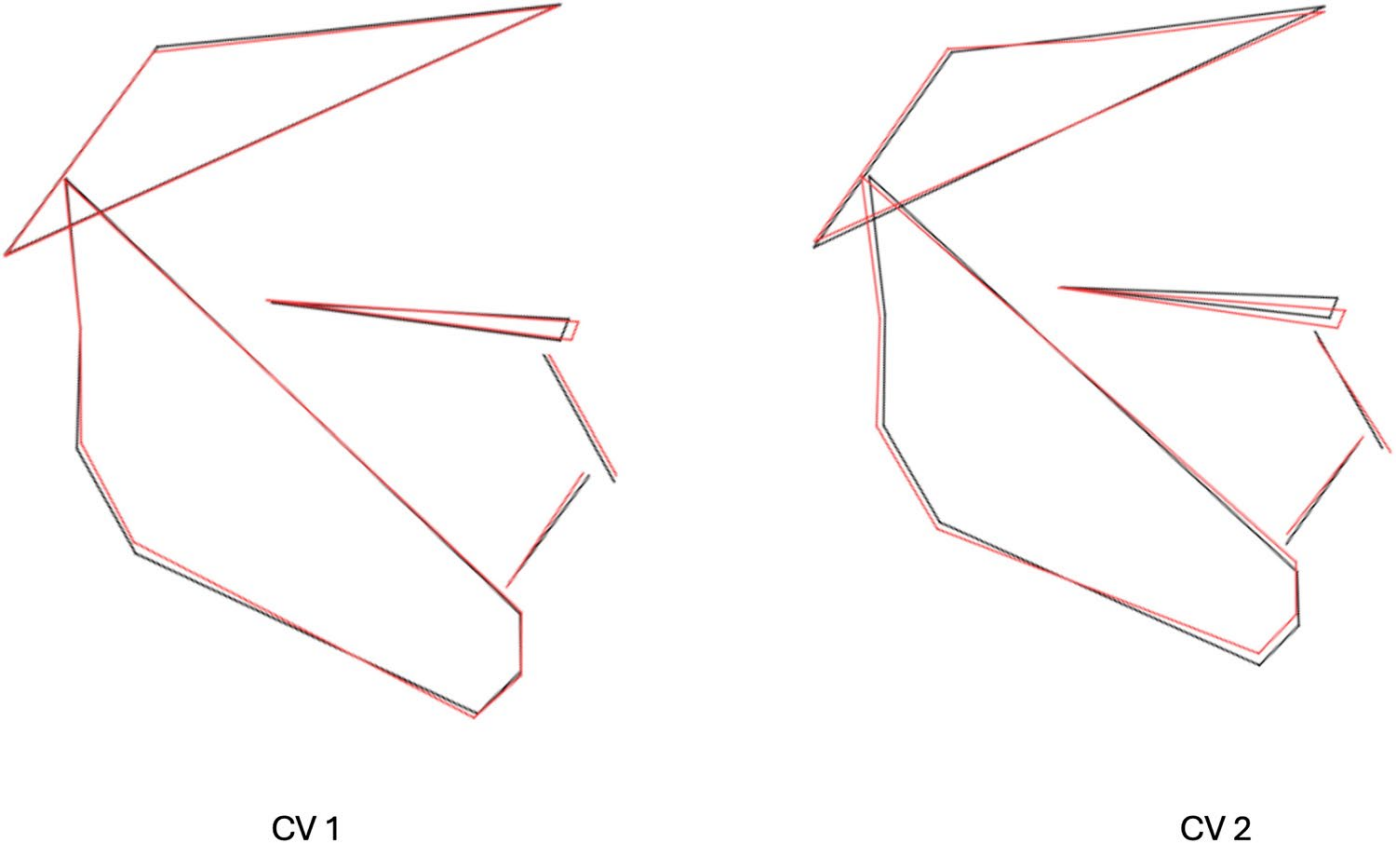
Wireframes depicting the mean shape (black color) and −3 standard deviation (SD) shape (red color) for the first two canonical variates (CV1 and CV2)

Pairwise comparisons (Table 1) revealed significant differences between subjects with and without clefts. The first canonical variate (CV1, 48% of variation) demonstrated that those differences were associated with maxillary shape and/or position and anterior and posterior facial height, while CV2 (34.8% of variation) pointed towards considerable differences in lower facial height (Fig. 3).

**Table 1:** Pairwise differences between groups in facial shape configurations assessed with canonical variates analysis (CVA)

	NORM		UCLA	
	Mahalanobis	Procrutes	Mahalanobis	Procrutes
UCLA	**1.8774**	**0.0332**		
	(< 0.001)	(< 0.001)		
UCLP	**2.1738**	**0.0277**	**1.451**	**0.0287**
	(< 0.001)	(< 0.001)	(< 0.001)	(< 0.001)

*P*-values (100,000 permutations) in brackets

### Craniofacial integration and modularity

In the NORM group, relatively high covariation, i.e., integration, was found between the skull base with the maxilla vs. the mandible, the skull base vs. the maxilla with the mandible, and the anterior vs. the posterior craniofacial compartments (Table 2), while in the scenarios involving the skull base with the mandible vs. the maxilla and the skull base vs. the maxilla vs. the mandible, the covariation was lower, implying the presence of modules. In three scenarios, patients with clefts exhibited the following covariation: the UCLAP group demonstrated the largest difference, and the UCLA group demonstrated an intermediate difference compared to the NORM group, while in 2 scenarios, the covariation patterns of skull base vs. maxilla vs. mandible and skull base with maxilla vs. mandible were less regular.

**Table 2:** Summary of analysis of integration of modularity with the multi-RV coefficient and covariance ratio

Number of modules	Skeletal parts in modules		(Multi-)RV coef			Covariance ratio†		
			*NORM*	*UCLA*	*UCLP*	*NORM*	*UCLA*	*UCLP*
3	Skb, Mx, Mn	coefficient	**0.325**	0.528	0.399	**0.779**	0.939	0.976
		p value	0.002	0.216	0.21	0.001	0.021	0.036
2	Skb + Mx, Mn	coefficient	0.597	0.566	0.603	1.053	1.053	1.080
		p value	0.441	0.897	0.559	0.405	0.364	0.396
2	Skb + Mn, Mx	coefficient	**0.248**	0.376	0.468	**0.650**	**0.839**	0.935
		p value	0.019	0.241	0.593	< 0.001	0.008	0.047
2	Skb, Mx + Mn	coefficient	0.535	0.498	0.427	1.027	1.060	1.005
		p value	0.341	0.729	0.188	0.244	0.279	0.137
2	Counterparts	coefficient	0.528	**0.378**	**0.403**	0.995	**0.831**	**0.875**
		p value	0.13	0.031	0.023	0.073	0.003	0.004

^†^For covariance ratio *p* < 0.01denotes statistical significance after Bonferroni correction

The results of testing the hypothesis of modularity, listed in Table 2 and the Appendix, demonstrated differences between subjects with and without clefts. The RV coefficient for the subdivision into 3 modules—the skull base, the maxilla, and the mandible—was significant for the NORM group (*p* < 0.05, Table 2). The 2-module subdivision—the skull base with the mandible vs. the maxilla—was also present in the NORM group, while the 2-module hypothesis representing the counterpart principle was accepted only in the UCLA and UCLAP groups. The CR after Bonferroni correction showed the same modularity pattern as revealed with the RV coefficients except for the subdivision representing the complex of the skull base-mandible vs. maxilla for the UCLA (*p* < 0.01, Table 2, Appendix), which suggested the presence of modularity in the UCLA group, whereas the RV coefficient failed to demonstrate modularity.

## Discussion

Craniofacial integration and modularity refer to the organization and development of the craniofacial complex. Although related, these concepts highlight different aspects of craniofacial development. Integration emphasizes the interdependence and coordinated development of craniofacial parts, whereas modularity implies the semi-independent nature of different craniofacial units, allowing for localized variation and adaptation. [6] In this study, we tested the research hypothesis that in unilateral orofacial clefts a different modularity pattern is present than in non-cleft controls.

We found that the modularity of the craniofacial region in unoperated adults with unilateral clefts—either UCLA or UCLAP—differs significantly from that in subjects without clefts. The primary difference lies in the presence of three distinct modules (skull base, maxilla, mandible) in unaffected controls, a pattern not observed in the cleft groups. Conversely, the UCLA and UCLAP groups exhibited a modularity pattern characterized by anterior and posterior modules, a configuration not observed in the NORM group. In the UCLA group, a 2-module scenario with the skull base and mandible forming one module and the maxilla forming another was suggested. However, this pattern was confirmed only by the CR and not by the RV coefficient, making it less conclusive. Our findings align with those of [18] despite methodological differences between the two studies. Richtsmeier and Delaeon (2009) assessed craniofacial integration in infants with UCLAP using 3D radiographic images while our study focused on craniofacial modularity in unoperated adults using 2D cephalograms. Richtsmeier and Deleon reported a correlation of 0.44 between the skull base and the face (equivalent to the maxilla and the mandible forming one module in our study) in infants with UCLAP, compared to 0.58 for unaffected controls. In our sample, the RV coefficient for the skull base vs the maxilla with mandible 2-module scenario was 0.43 for the UCLAP group, 0.5 for the UCLA group, and 0.54 for unaffected controls. The presence of anterior and posterior modules in subjects with clefts supports the concept of an anterior and posterior craniofacial column [19]. These columns can be seen as two vertically oriented modules, each comprising highly integrated submodules. The anterior column includes the anterior skull base, ethmomaxillary complex, and mandibular corpus, while the posterior column comprises the posterior skull base and mandibular ramus [19]. Why the counterpart principle was not detected in the NORM group will be discussed later. Since no other study has investigated craniofacial integration and modularity in patients with orofacial clefts, further comparisons are not possible.

The present findings imply that craniofacial integration and modularity differ between our Indonesian controls of Proto-Malayid ethnicity and those of European ethnicity [3, 5]. Using a similar methodology proposed by Klingenberg [6] with an adjacency graph constructed using Delaunay triangulation and the RV coefficient to identify modularity patterns, Wellens et al. identified two 2-module scenarios—the skull base vs. the maxilla with the mandible and the counterpart scenario (i.e., the anterior vs. posterior module)—in their noncleft sample comprising 179 orthodontic patients. In our noncleft sample, we found 3 relatively independent modules: the skull base, the maxilla, and the mandible. We also confirmed an alternative 2-module scenario in which the skull base and mandible formed one module and the maxilla formed another module.

Several factors could explain this difference. First, our noncleft control group was smaller than that examined by Wellens et al. (50 vs. 179 subjects), which may influence the value of the RV coefficient [17, 20]. Second, we used a slightly different set of landmarks for identifying craniofacial structures, particularly the skull base. For example, we did not use the Porion and Orbitale landmarks, which are difficult to identify, but we included the landmark Posterior ethmoidale. This may have resulted in different covariation patterns of craniofacial regions and hence different modularity patterns.

Finally, it is possible that the organization, integration, and modularity of craniofacial regions differ between European and Asian populations. Although no direct comparisons have been published, indirect epidemiologic evidence suggests significant differences. For example, there is a relatively frequent occurrence of Angle Class III malocclusion with mandibular prognathism/maxillary retrognathism in Asia compared to Europe [21, 22]. Craniofacial features of Class III malocclusion, such as a decreased saddle angle (i.e., the angle between anterior and middle cranial fossae) or increased gonial angle, are established early in ontogeny [23], and subsequent growth makes them more pronounced [24]. This is likely associated with increased covariance between the skull base and the mandible, leading to the formation of a module. The relatively high prevalence of Class III features in Asian populations, including the Indonesian population from which our sample was derived, could create a modularity signal detected in our study.

A final possible explanation for the tenfold higher RV and CV values in unoperated adults with clefts compared to controls, is that the integration and modularity patterns in these unoperated individuals may revert to more basic, life preserving patterns. In these individuals, the posterior module likely remains highly integrated to ensure airway and pharyngeal patency [25]. In contrast, the functionally healthier control group exhibits more developmental flexibility or ‘’headroom’’ for local integration across multiple craniofacial modules.

We used two measures of covariation—the RV coefficient and the CR—because the RV coefficient has several shortcomings. Specifically, the RV coefficient can be influenced by sample size and the number of variables, which can confound the results. This confounding effect can limit the biological interpretations that can be drawn from the RV coefficient and make comparisons of RV coefficients across different datasets less reliable [17]. The CR, proposed to overcome these limitations of the RV, has been shown to be a robust method for quantifying modularity in data and evaluating it relative to the null hypothesis of no modular structure [26]. In our study, however, the RV and CR showed similar behavior except in the 2-module scenario where the skull base and mandible form one module and the maxilla forms another in the UCLA group. The RV indicated no presence of such modules, whereas the CR confirmed the opposite, albeit not strongly.

The observed patterns of craniofacial modularity in individuals with unoperated clefts have significant clinical implications for treatment planning and postoperative outcomes. The distinct anterior–posterior modular organization in cleft-affected individuals, compared to the more integrated skull base-maxilla-mandible structure in unaffected controls, suggests that surgical interventions should account for these inherent developmental differences. Recognizing these variations could help refine treatment strategies by anticipating how different craniofacial regions may respond to surgery and postoperative remodeling. For example, understanding the modular separation by the pterygomaxillary plane in individuals with clefts may inform surgical sequencing, minimizing unintended structural compensations. Additionally, these insights highlight the importance of individualized treatment approaches that consider congenital craniofacial organization, potentially improving long-term functional and aesthetic outcomes.

### Limitations

As mentioned in the data analysis section, we encountered difficulties in maintaining a constant radiographic magnification factor resulting the study was done solely on facial shape. The soft-tissue contours were also difficult to identify, so the analysis of facial morphology was limited to hard tissues. We assessed craniofacial integration and modularity in groups comprising both males and females because previous studies have not shown significant sex differences in craniofacial organization. Nevertheless, we cannot entirely exclude the possibility of sexual dimorphism in our sample. Additionally, we did not evaluate the allometric effect of size on integration and modularity in our sample because it was not possible to correct for differences in magnification on cephalograms (i.e., it was not possible to establish the size). Although studies have shown a small effect of allometry on craniofacial organization [3], this remains a potential confounding factor in our analysis. In our study, we compared an untreated group with a non-cleft control group. It opens the possibility for future research comparing a group of patients with the same ethnic background who have had surgery.

## Conclusions

The findings of this study suggest that untreated UCLA and UCLAP affect craniofacial integration and modularity:

- Individuals with clefts exhibit a distinct anterior–posterior modular organization divided by the pterygomaxillary plane, whereas controls show separate modules for the skull base, maxilla, and mandible.
- The greatest craniofacial shape differences were observed between UCLAP and unaffected controls, with UCLA and UCLAP groups being more similar to each other.
- Vertical and sagittal shape variation were the primary drivers of craniofacial differences among groups.
- Findings underscore the importance of considering congenital craniofacial organization when assessing treatment outcomes, as cleft-affected individuals may exhibit different surgical responses compared to controls.

## Supplementary Information

Below is the link to the electronic supplementary material.

Supplementary file1 (DOCX 242 KB)

## Data Availability

Data files can be obtained from the corresponding author upon reasonable request.
