# Supplementary material for: Craniofacial integration and modularity in untreated cleft lip and palate
Source: Clin Oral Investig. 2025 Mar 31;29(4):218. doi: 10.1007/s00784-025-06296-3 (PMC11958437; doi:10.1007/s00784-025-06296-3)

**APPENDIX**

**Craniofacial integration and modularity in untreated cleft lip and palate**

**Supplementary Table 1.** Results of Principal Component Analysis (PCA)

| **Principal components** | **Eigenvalues** | **% Variance** | **Cumulative %** |
| --- | --- | --- | --- |
| 1. | 0.00139515 | 26.2 | 26.2 |
| 2. | 0.00082574 | 15.5 | 41.7 |
| 3. | 0.00066939 | 12.6 | 54.3 |
| 4. | 0.00036516 | 6.9 | 61.1 |
| 5. | 0.00030094 | 5.7 | 66.8 |
| 6. | 0.00027566 | 5.2 | 72 |
| 7. | 0.00023383 | 4.4 | 76.4 |
| 8. | 0.00017274 | 3.2 | 79.6 |
| 9. | 0.00015551 | 2.9 | 82.5 |
| 10. | 0.00014832 | 2.8 | 85.3 |
| 11. | 0.00011443 | 2.1 | 87.5 |
| 12. | 0.00011087 | 2.1 | 89.5 |
| 13. | 0.00010474 | 2 | 91.5 |
| 14. | 0.00007924 | 1.5 | 93 |
| 15. | 0.00007666 | 1.4 | 94.4 |
| 16. | 0.00006195 | 1.2 | 95.6 |
| 17. | 0.00005273 | 1 | 96.6 |
| 18. | 0.00005106 | 1 | 97.5 |
| 19. | 0.00003754 | 0.7 | 98.2 |
| 20. | 0.00003069 | 0.6 | 98.8 |
| 21. | 0.00002502 | 0.5 | 99.3 |
| 22. | 0.00001684 | 0.3 | 99.6 |
| 23. | 0.00001253 | 0.2 | 99.8 |
| 24. | 0.00000836 | 0.2 | 100 |

**Supplementary Table 2.** Results of analysis of integration of modularity with the multi-RV coefficient


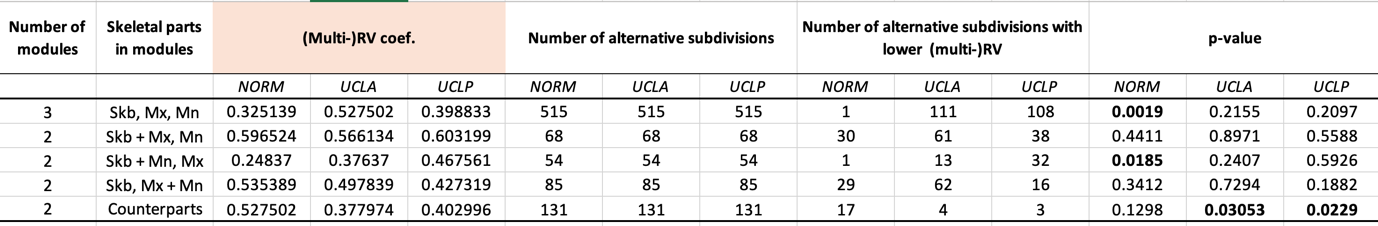


**Supplementary Table 3.** Results of analysis of integration of modularity with the covariance ratio


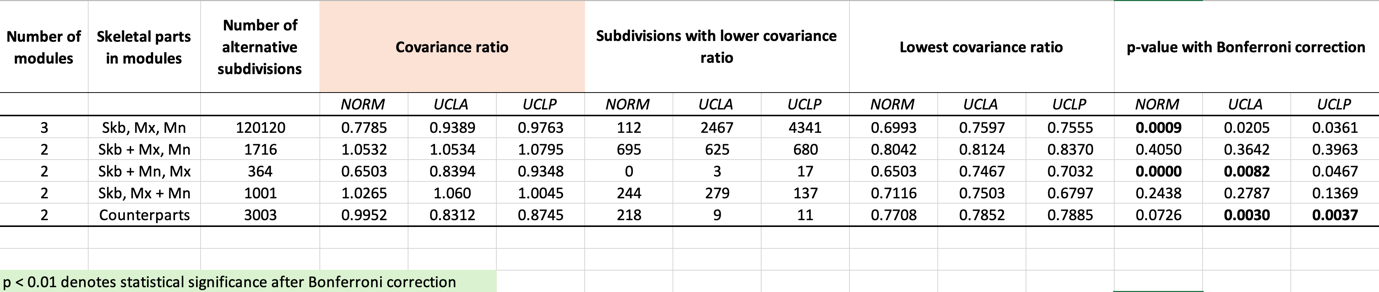

Supplement: Supplementary file 1 — Supplementary file1 (DOCX 242 KB) [file 784_2025_6296_MOESM1_ESM.docx]
